# Color-selective photodetection from intermediate colloidal quantum dots buried in amorphous-oxide semiconductors

**DOI:** 10.1038/s41467-017-00893-x

**Published:** 2017-10-10

**Authors:** Kyung-Sang Cho, Keun Heo, Chan-Wook Baik, Jun Young Choi, Heejeong Jeong, Sungwoo Hwang, Sang Yeol Lee

**Affiliations:** 1 Samsung Advanced Institute of Technology, Device Lab, Suwon, 16678 South Korea; 2 Samsung Advanced Institute of Technology, Research Center for Time-Domain Nano-Functional Device, Suwon, 16678 South Korea; 3 School of Electrical Engineering, Korea University, Seoul, 02841 South Korea; 4 Department of Semiconductor Engineering, Cheongju University, Cheongju, 28503 South Korea; 5 Present Address: Department of Physics and Institute for Advanced Study, The Hong Kong University of Science and Technology, Clear Water Bay, Hong Kong, China

## Abstract

We report color-selective photodetection from intermediate, monolayered, quantum dots buried in between amorphous-oxide semiconductors. The proposed active channel in phototransistors is a hybrid configuration of oxide-quantum dot-oxide layers, where the gate-tunable electrical property of silicon-doped, indium-zinc-oxide layers is incorporated with the color-selective properties of quantum dots. A remarkably high detectivity (8.1 × 10^13^ Jones) is obtained, along with three major findings: fast charge separation in monolayered quantum dots; efficient charge transport through high-mobility oxide layers (20 cm^2^ V^−1^ s^−1^); and gate-tunable drain-current modulation. Particularly, the fast charge separation rate of 3.3 ns^−1^ measured with time-resolved photoluminescence is attributed to the intermediate quantum dots buried in oxide layers. These results facilitate the realization of efficient color-selective detection exhibiting a photoconductive gain of 10^7^, obtained using a room-temperature deposition of oxide layers and a solution process of quantum dots. This work offers promising opportunities in emerging applications for color detection with sensitivity, transparency, and flexibility.

## Abstract (web summary)

The development of highly sensitive photodetectors is important for image sensing and optical communication applications. Cho et al., report ultra-sensitive photodetectors based on monolayered quantum dots buried in between amorphous-oxide semiconductors and demonstrate color-detecting logic gates.

## Introduction

The detection of light has historically been one of the most fundamental and academic subjects behind various optoelectric applications^1–11^. At present, the development of photodetectors, which convert incident photons to electrical signals, has confronted significant challenges regarding the realization of efficient and sensitive detection with low noise for the ultraviolet (UV)^3, 4^, visible^5–9^, and infrared regimes^10, 11^ of electromagnetic spectrum. Furthermore, a strong demand for complementary metal-oxide semiconductor (CMOS)-compatible, monolithic integration with a low-cost and simple fabrication process has arisen, which may not be achievable using epitaxially grown III–V semiconductors for photoconductors, avalanche photodiodes, and photomultipliers. Such devices have disadvantages related to material growth conditions, high-voltage operation, and bulkiness, including increased fabrication cost and complexity. However, colloidal quantum dots (QDs), often referred to as semiconductor nanocrystals, are easily processible for integration onto various substrates using a low-cost, solution-coating method. They have unique optical properties, such as bandgap energies tunable by adjusting their sizes, narrow emission bandwidths, broad absorption spectrum, and high photoluminescence quantum efficiencies. These outstanding advantages have incited considerable research efforts toward the development of QD photodetectors^1–11^, which now target the performance of conventional state-of-the-art photodetectors.

One of the figures of merit characterizing photodetection is the normalized detectivity (D*), expressed in units of Jones (cm Hz^1/2^ W^−1^), equal to the square root of the optically active area divided by its noise equivalent power (NEP). Konstantatos et al.^5^ have demonstrated visible and near-infrared (NIR)^10^ photoconductive detectors using PbS colloidal QDs, and reported D* values of 5 × 10^12^ and 1.8 × 10^13^ Jones, respectively. Their achievement in the visible spectrum is comparable to the results for conventional silicon photodiodes ( ~ 4 × 10^12^ Jones)^2^, because of the relatively high mobility (10^−2^ ~ 1 cm^2^ V^−1^ s^−1^) of the functionalized PbS QDs caused by the large wavefunction overlap. Lee et al.^6^ have reported visible photoconductors consisting of CdSe colloidal QDs capped with an In_2_Se_3_ metal chalcogenide complex, and achieved a D* of 1 × 10^13^ Jones, contributed by the high-mobility (16 cm^2^ V^−1^ s^−1^). The purpose of that study was to obtain high mobility via ligand exchange, due to the intrinsically low mobility of QDs (10^−6^ cm^2^ V^−1^ s^−1^) for visible detection^12, 13^.

In contrast to photoconductor devices, phototransistors provide a wider degree of photocurrent control, which is achievable by adjusting the gate voltages (*V*_G_) as well as the source-drain voltages and incident light intensities. Recently, QD-hybrid phototransistors have been introduced for use in combination with high-mobility materials, e.g., amorphous-oxide semiconductors (AOSs)^7–9, 14^, graphene^11^, and 2-D materials^15, 16^. PbS colloidal QDs on mechanically exfoliated graphene flakes, for example, have exhibited device D* values as high as 7 × 10^13^ Jones, with a responsivity (*R*) of 10^7^ AW^−1^ ^11^), the ratio of the generated photocurrent to the incident optical power. The high electron mobility (c.a. 1000 cm^2^ V^−1^ s^−1^) of graphene contributes to the fast hole transfer, while the electrons remain in trap states. However, interface control between QDs and graphene remains an issue^2, 17^.

On the other hand, AOS materials with wide bandgap energies have attracted particular attention in the fields of electronics and optoelectronics^4, 18–23^. The advantages of AOS films are their high transparency, low processing temperature, smooth surface with no grain boundary, and high electron mobility regardless of the degree of film disorder^18–21^. The application of the majority of AOS materials to visible wavelength region remains challenging, because of their intrinsically wide bandgaps. That is, the use of AOSs for photodetection is, in principle, limited to UV energy band^4, 22^. However, continuous attempts have been made to use AOSs in image sensors or phototransistors, in parallel with the development of active-matrix thin-film transistors (TFTs) for use in display devices^18, 23^, to replace amorphous or poly silicon. Recently developed AOS films as channel materials in TFTs demonstrated high electron mobilities of more than 30 cm^2^ V^−1^ s^−1^ ^18^). For example, In-Ga-Zn-O (IGZO) and Ge-doped In-Ga-O (Ge-IGO) layers have been used as high-mobility channels in conjunction with CdSe QDs located on either the top or bottom surfaces of the oxide layers^7–9^. In the channel configuration of QDs on oxide (QO), i.e., QDs on IGZO, *R* reached an order 10^4^ AW^−1^ in UV range, while a photocurrent saturation was observed for an input light intensity of more than 300 μW cm^−2^ ^7^). In this case, the charge separation from QDs to AOS is considered to be dependent on the degree of contact between QD and-AOS. In addition, phototransistors with PbS QDs on IGZO demonstrated a photoresponsivity of 10^6^ AW^−1^ in NIR range^14^. Thus, the demonstration of photodetection using QDs with AOS offers promising opportunities, including potentially promising multispectral photodetection in visible spectrum, but has yet to yield high *R* and D*.

Here, we propose a hybrid QD-AOS phototransistor composed of an oxide-QD-oxide (OQO) layered channel, in which the intermediate QDs are buried in between the top and bottom oxide layers. This may provide a fast charge separation from QD to AOS layer by the increase of interface area between QD and AOS. Amorphous SIZO (Si-doped In-Zn-O) films are chosen to allow room-temperature, radio-frequency (RF) sputtering and low-temperature, annealing processes^20, 21^. We employ CdSe, CdSeS, and CdS QDs for red (R), green (G), and blue (B) color detection, respectively, using their particular absorption characteristics. As an example of color-selective detection, we demonstrate a logic circuit comprised of an electro-optical NOT gate using different wavelength illuminations. This hybrid OQO configuration is expected to achieve highly sensitive photodetection because of fast charge separation induced by the large interfacial areas of QDs buried in the oxide layers, along with the fast carrier transport caused by the high mobility of the oxide layers. In particular, a rigorous characterization of D* with respect to the noise spectrum is conducted as a function of modulation frequency for the proposed hybrid OQO phototransistors.

## Results

### Photoresponse of hybrid QD-AOS phototransistors

**Fig. 1:**
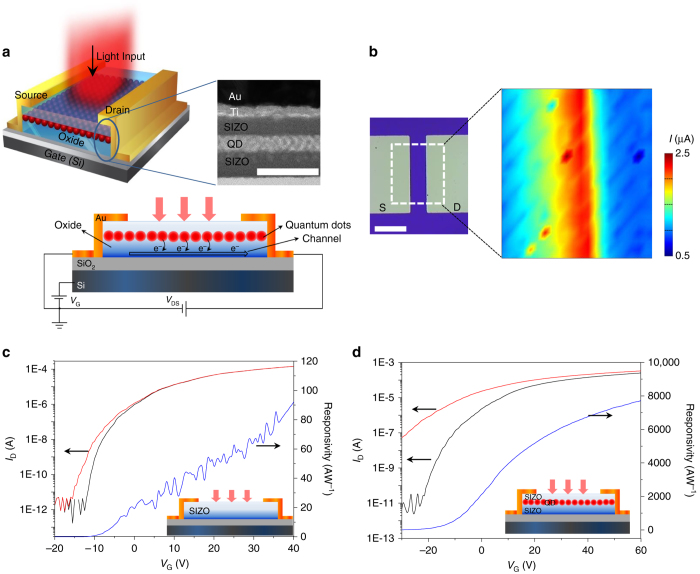
Hybrid phototransistors of quantum dots buried in amorphous-oxide semiconductors. **a** Schematics of proposed device structure. The channel contains OQO layers, with QDs buried between the *top* and *bottom* oxide (SIZO) layers, as shown in the cross-sectional TEM image. *Scale bar* is 100 nm. The *top* and *bottom* SIZO layer thicknesses are 30 and 40 nm, respectively. The 1–6 QD layers have 5.5–30-nm thicknesses. The photocarriers generated by the QDs via light absorption are transported through the oxide layers. **b** Spatial photocurrent profile for 487-nm laser beam with 10-nW power. *Scale bar* is 100 nm. The photocurrent was measured during laser beam scanning across the photodetector surface. **c**, **d** Photocurrent response and *R* of (**c**) SIZO-only device and (**d**) monolayered-red-QD (1RQ) OQO device, at 487-nm illumination wavelength with 10-nW power. *V*_SD_ is 5 V. *Black* and *red lines* are dark current and photocurrent, respectively. Responsivities are indicated by *blue lines*

A schematic of the proposed hybrid OQO phototransistor is shown in Fig. 1. The OQO layer is located between the source and drain electrodes, to absorb the incident light, generate photocarriers, and to transport the carriers to the electrodes under the control of the back-gated voltages. The transmission electron microscopy (TEM) image (Fig. 1a, *inset*) shows the spin-coated QD layer buried in between the top and bottom SIZO layers. We chose SIZO as a channel material because it exhibits high mobility in the amorphous phase via controlled doping of silicon in the IZO. An electron mobility greater than 20 cm^2^ V^−1^ s^−1^ is provided by the room-temperature RF-sputtered SIZO layers, following a 150 °C annealing process^20^. In contrast, an annealing temperature of more than 300 °C is typically required for other oxide materials, e.g., In-Ga-Zn-O, Zn-Sn-O, and In-Ga-O^18^. We measured the mobilities of the OQO films for R, G, and B QDs buried in the SIZO layers and for different QD layer thicknesses, along with that of a 40-nm SIZO layer without QDs (see Supplementary Table 1). Hence, we found that the Ds buried in the SIZO layers might not affect the SIZO channel mobility. Figure 1b shows the photocurrent mapping of the OQO phototransistor, indicating that the photocarrier generation occurs across the entire OQO area, not from the electrodes or their edges. Figure 1c, d show the transfer characteristics, together with the *R* behaviors of a single-layered SIZO transistor and a monolayered-red-QD OQO (1RQ-OQO) transistor, respectively, under dark and light irradiation. In the SIZO-single-layered device, there is almost no absorption of input light at 487-nm wavelength because of the wide bandgap energy of 3.2 eV^24^) (see Supplementary Fig. 2). On the contrary, the measured photocurrent of the OQO device increases dramatically via photocarrier generation owing to the QD light absorption, as depicted in Fig. 1d. The *R* of the SIZO-single-layered device without QDs is less than 100 AW^−1^; however, this value increases to more than 6000 AW^−1^ in the 1RQ-OQO device, where *R* = (*I*_SD, light_ − *I*_SD, dark_)/*P*_light_ (AW^−1^), where *I*_SD, light_ is the drain current under irradiation of light, *I*_SD, dark_ is the drain current under dark, and *P*_light_ is the irradiation light power^2^ (see also Supplementary Fig. 3 as depicted in linear scale).

### Photocarrier generation and transport

**Fig. 2:**
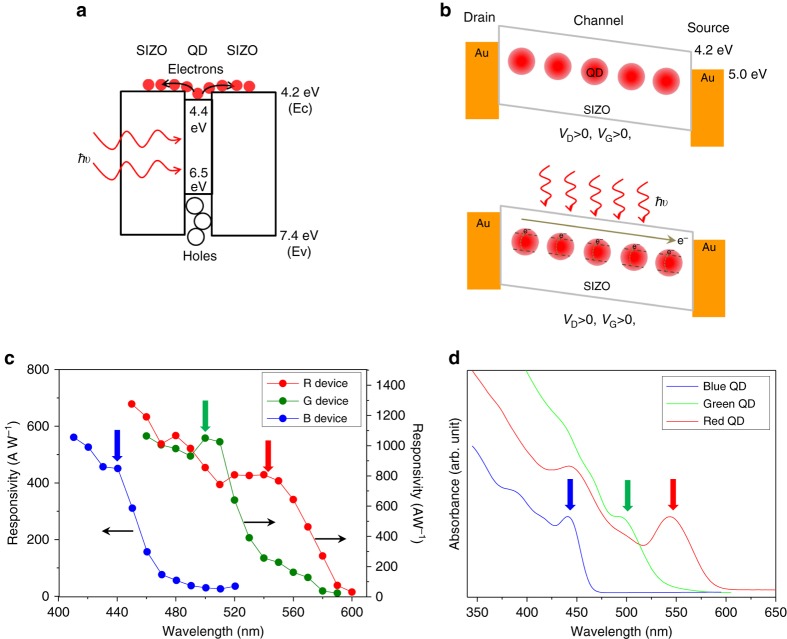
Physical principles and spectral responses. **a** Energy diagram of red-QD OQO structure. **b** Light-induced carrier generation and transport mechanisms. **c** Spectral responses of 4RQ, 6GQ, and 6BQ OQO phototransistors. **d** Absorption spectra of red, green, and blue QDs

The energy-band diagram and the operating principles of the OQO phototransistors are shown in Fig. 2a, b, respectively. The bandgap and band positions of the QD and SIZO are determined from the measurement of ultraviolet/visible (UV/Vis) absorption spectrum, UV photoelectron spectroscopy, electron energy loss spectrum, and Kelvin probe method^21^. The QD Fermi level, 4.4 eV, is slightly higher than that of SIZO, 4.2 eV, which allows charge separation via QD light absorption. The photocarriers generated by the charge separation flow with respect to the potential difference (*V*_D_) between the source and drain electrodes under the control of *V*_G_, as illustrated in Fig. 2b. Figure 2c shows the color spectral *R* trends of phototransistors with 4-layered-red-QD OQO (4RQ-OQO), 6-layered-green-QD OQO (6GQ-OQO), and 6-layered-blue-QD OQO (6BQ-OQO) configurations (see also, Supplementary Fig. 4). The photocurrent response for each color follows the QD absorption, as shown in Fig. 2d. This implies that the photoresponse of the OQO phototransistors comes from the RGB photoexcitation of the QDs, rather than the defect-level-mediated carrier excitation of the SIZO. Because the SIZO bandgap energy (3.26 eV) is larger than that of the QDs for visible spectrum, various kinds of QDs are applicable to the proposed OQO structure. Although QDs mostly have broad absorption spectrum, their color-dependent photoresponse provides spectral information as well by appropriate signal processing method^25^. Therefore, our OQO phototransistors can be usefully implemented in color-selective photodetection.

### Characteristics in monolayered-QD OQO configuration

**Fig. 3:**
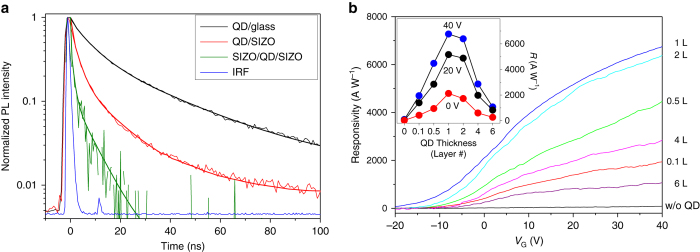
Measurement results of fluorescence decays and photoresponses. **a** Fluorescence decay curves of three different monolayered-red-QD films, i.e., QDs, QDs on SIZO, and SIZO/QD/SIZO (OQO), all on glass substrates. The *blue line* is the system instrumental response function (IRF). The thicker *solid lines* are the fitting of each decay *curves*. **b** *R* vs. *V*_G_ with respect to QD layer thickness for red-QD OQO phototransistors. The incident light at 487-nm wavelength is illuminated with 10-nW power. In all, 0.1 and 0.5 L correspond to areal coverage of 10% and 50% of the monolayered-QD films, respectively. The *inset* figure shows the *R* values vs. the QD layer thickness

Figure 3a shows the fluorescence decay curves of three different monolayered-red-QD films (QD on glass, QD on SIZO, and SIZO/QD/SIZO, i.e., OQO). The fluorescence decay time of the QD on SIZO is shorter than that of the QD on glass, because of the fast charge separation from the QDs to the SIZO film. The charge separation rate is calculated to be approximately 0.5 ns^−1^. Furthermore, the charge separation rate of the OQO film increases up to 3.3 ns^−1^, corresponding to more than six times that of the QD-on-SIZO film (see Supplementary Method 2). This remarkable improvement stems from the increment of the effective interfacial area for the QDs buried in the amorphous SIZO films, i.e., the OQO structure, compared with the single-side contact experienced by the QDs on the SIZO film. The detailed discussion on the interfacial area is presented in Supplementary Fig. 5 and Supplementary Note 1. We further prepared and compared the photocurrent response of QO devices, i.e., monolayered-red-QD QO (1RQ-QO) phototransistors. They did not show a remarkable increase of *R* compared to OQO configuration (see Supplementary Fig. 6).

We measured the *R* of the proposed OQO structure vs. *V*_G_ with respect to the QD layer thickness, as depicted in Fig. 3b. The QD layer thickness varied from zero to six layers. In this figure, the “0.1 L” and “0.5 L” QD layers are sub-monolayered QD films, with areal coverages of 10% and 50% monolayered-QD films, respectively, (see Supplementary Figs. 7 and 8 for TEM and scanning electron microscope images for QD films). When the thickness increases up to one (mono) layer, i.e., the areal coverage becomes 100%, the *R* increases steadily at fixed *V*_G_; however, it begins to decrease once multiple layers are added. As illustrated in the inset of Fig. 3b, where *R* is replotted against the QD layer thickness, the monolayered-QD OQO device exhibits the highest *R*. In our hybrid OQO phototransistors, the photoresponse mechanism can be described as follows: photocarrier generation via QD light absorption; charge separation from the QDs to the top and bottom SIZO layers; and carrier transport through the SIZO films to the electrodes. The photocurrent generation, which is proportional to the number of QDs, dominates up to monolayered-QD film structures; however, the charge separation rate and the carrier transport become important factors for 2-, 4-, and 6-layered QDs. In other words, the charge separation rate may not change, because the interfacial area between the QDs and their surrounding SIZO films does not effectively increase for structures with more than one-layer QD thickness. In particular, the photocarrier transport from QDs to the SIZO layers is thought to be disturbed significantly by the increasing number of charge trap sites in the multiple QD layer structures as discussed in the previous studies^26–30^. These have revealed low electronic conductivity in semiconductor nanocrystal arrays because of large concentrations of surface dangling bonds given by trap sites. More details are discussed in Supplementary Note 2. The above results show that the proposed hybrid OQO structure is superior to other configurations, when monolayered QDs are buried between the top and bottom amorphous SIZO layers.

### Device performance of monolayered-QD OQO phototransistors

**Fig. 4:**
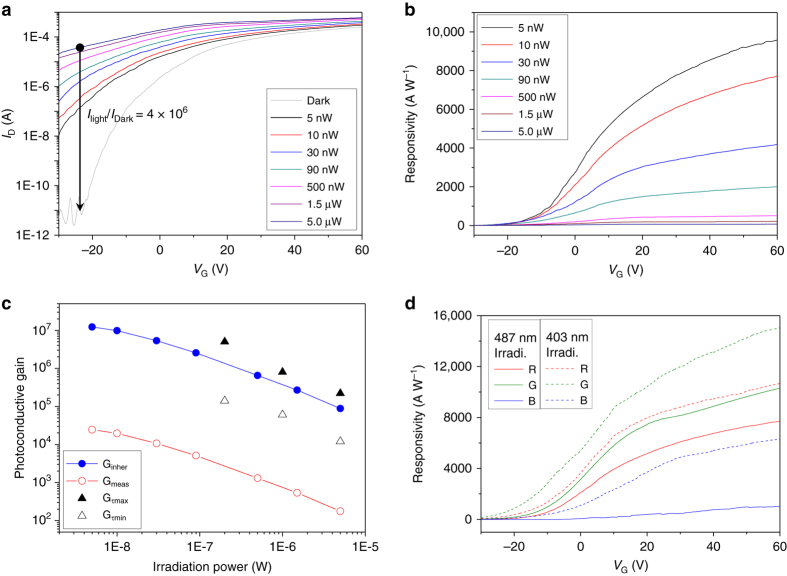
Photoresponse characteristics and photoconductive gains. **a** Photocurrent responses and **b** *R* trends of 1RQ-OQO phototransistors with respect to incident light power. **c** Photoconductive gains using three different estimation methods vs. incident light power. **d** *R* trends of 1RQ-, 1GQ-, and 1BQ-OQO phototransistors at 487- and 403-nm wavelengths with 10-nW power

Figure 4a, b show the transfer characteristics and *R* behavior of 1RQ-OQO devices as functions of *P*_light_ at 487-nm wavelength. The on-off ratio (*I*_light_/*I*_dark_) at *V*_G_ = −22 V is approximately 4 × 10^6^ (66 dB), as shown in Fig. 4a. We successfully detected a wide range of incident light powers, corresponding to a range of three orders of magnitude (5 nW–5 μW). Note that the 1RQ-OQO configuration does not exhibit photocurrent saturation, even at 5 μW (i.e., 255-mW cm^−2^ light intensity). This is one of the advantages of fast charge separation in OQO configuration. (see Supplementary Note 3). In contrast, a previous study of phototransistors with QDs on IGZO has reported saturation at a significantly lower light intensity of 300 μW cm^−2^ ^7^). In Fig. 4b, *R* is as high as 9800 AW^−1^ at 60-V *V*_G_, for a 5-nW incident light power (i.e., 255-μW cm^−2^ light intensity). This result confirms the highly sensitive response of the OQO device. We also found the inverse relation between *R* and the incident light power, which may be explained by filling of the lowest-lying, longest-lived trap states that provide the highest photoconductive gain (*G*_meas_) at low light intensities, as shown by Konstantatos et al.^5^.

Note that *G*_meas_ is one of the important figures of merit. This property is estimated from the ratio of the collected photocarrier rate at the electrodes and the incident photon flux, as given by *G*_meas_ = *R ·* *hυ*/*eη*, where *R* corresponds to the measured results s*h*own in Fig. 4b, *h* is the Planck constant, *υ* is the frequency of light, *e* is the electron charge, and *η* is the quantum efficiency. Figure 4c indicates that the *G*_meas_ for 1RQ-OQO phototransistors is in the 10^2^–10^4^ range at *V*_G_ = 60 V, with 487-nm illumination. However, we must also consider an inherent photoconductive gain (*G*_inher_), which includes the net absorption in the OQO channels, by eliminating the reflection (*R*_light_) and transmission (*T*_light_) of the devices^31^. Thus, *G*_inher_ = *G*_meas_/(1−*R*_light_−*T*_light_), because the majority of the photon flux is transmitted by the monolayered-QD configuration. Here, we obtained a *G*_inher_ of 10^5^–10^7^ for a light power of 5 μW–5 nW (see Supplementary Note 4). This implies that the photocarrier generation is highly efficient, even for a tiny amount of incident photons. We further investigated and compared the photoconductive gains using the photoresponse decay time (*τ*_lifetime_) and the carrier transit time (*τ*_transit_) of the OQO device, where *G*_*τ*_ = *τ*_lifetime_/*τ*_transit_. The measured decay time can be represented by two components with fast and slow decays using a two-exponential fitting method, as shown in Supplementary Fig. 11 (see also Supplementary Note 5). Furthermore, *τ*_transit_ = *L* ^2^/*μ* · *V*_SD_, where *L* is the length between the source and drain electrodes, *μ* is the channel mobility, and *V*_SD_ is the voltage difference between the electrodes. Here, *τ*_transit_ = 5.0 × 10^−7^ s, obtained using *L* = 50 μm, *μ* = 10 cm^2^ V^−1^ s^−1^, and *V*_SD_ = 5V. Our prediction for *G*_*τ*_ is located in the 1.2 × 10^4^–2.2 × 10^5^ range, as indicated by the empty and filled triangles in Fig. 4c, for an irradiation power of 5 μW, for example. Therefore, this estimation of the upper and lower bounds of *G*_*τ*_ using the fast and slow decay times indicates that the use of *G*_inher_, considering the net absorption, is both practical and accurate for our OQO configuration, because *G*_inher_ is situated midway between the upper and lower bounds of *G*_*τ*_.

Based on these results, we prepared three different OQO phototransistors with monolayered QDs for R, G, or B colors. We chose 403- (near UV) and 487-nm (sky blue) illumination wavelengths as sample cases, to investigate the color-selective detection of the three devices. Figure 4d shows that the 403-nm irradiation on the 1RQ- and 1GQ-OQO phototransistors yields higher *R* than the 487-nm illumination; this is because of the larger absorption, as discussed with regard to Fig. 2d. The *R* of the 1GQ-OQO phototransistor reaches 14,900 AW^−1^ at a *V*_G_ of 60 V. The response of the 1BQ-OQO device is notable in that it is activated at 403-nm wavelength, but there is almost no response for the 487-nm illumination; this is because of the lack of absorption. This result suggests highly sensitive RGB-color-selective detection when the *R* threshold levels are chosen appropriately at the desired wavelength of illumination.

### Logic inverter using color-selective detection

**Fig. 5:**
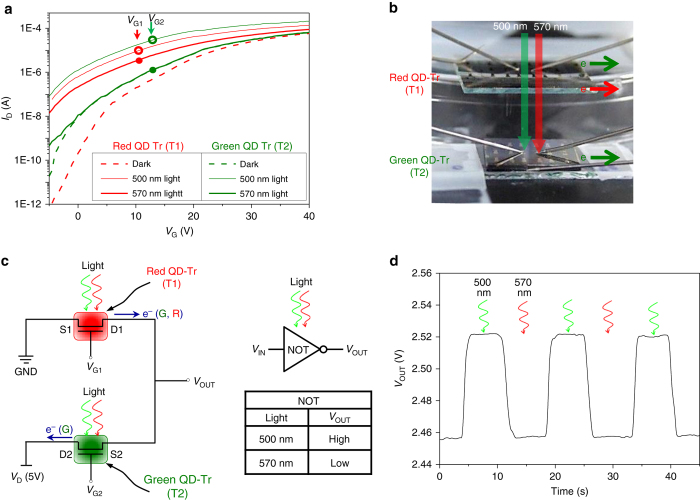
Logic inverter using red- and green-quantum dot phototransistors. **a** Photoresponses of 1RQ- and 1GQ-OQO phototransistors with respect to incident light wavelengths. Both transistors exhibit photoresponses at 500-nm wavelength illumination. For 570-nm-wavelength irradiation, the photoresponse is only observed for the 1RQ-OQO devices. **b** Stacked configuration of 1RQ- and 1GQ-OQO phototransistors prepared on transparent glass substrates for logic inverters. **c** Electro-optical NOT gate circuitry and resultant logic table. **d** Time-domain measurement of *V*_out_ at alternated 500- and 570-nm illumination wavelengths

Figure 5 shows the logic circuit of an electro-optical NOT gate (inverter) as a simple embodiment of color-selective detection. Taking into consideration the flexibility in the control of parameters such as the QD absorption energy band, the illumination wavelength with power, and *V*_G_, we employed 1RQ- and 1GQ-OQO phototransistors for color-selective detection. Each transfer curve was measured at two different illumination wavelengths, 500 and 570 nm, as depicted in Fig. 5a. From these curves, an operating condition for a NOT gate that outputs a voltage representing the opposite logic level to its input can be chosen. We selected the gate voltages (*V*_G1_ = 10 V and *V*_G2_ = 12 V) indicated by the colored circles and filled dots. Figure 5b shows a stacked configuration of 1RQ- and 1GQ-OQO phototransistors (T1 and T2, respectively), which has the advantage of transparent OQO channels (see Supplementary Note 6) for light illumination. When devices are illuminated with light of 500- or 570-nm wavelength, the read-out voltage (*V*_OUT_) outputs high or low levels between the source and drain electrodes of each device, respectively. At 500-nm irradiation, the voltage drop across T1 is larger than that across T2, because of the higher impedance of T1, as shown in Fig. 5a. On the contrary, *V*_OUT_ at 570 nm becomes low because of the small impedance of T1, which is due to the large photo-induced charges in the T1 channel. These results are summarized in the table included in Fig. 5c and correspond to a standard NOT gate. Therefore, we successfully demonstrated the on-and-off switching characteristics of *V*_OUT_ using two different illumination wavelengths in the time domain, as shown in Fig. 5d. We may further improve the response time using fast-switching optical sources, because here we employed optical parametric oscillator (OPO) laser system (Opolette HR 355, OPOTEK) requiring 2.3 ~ 2.4 s for switching the wavelengths in high accuracy. This kind of logic circuits for color-selective detection provides opportunities for color-filterless photodetection. CMOS image sensors, for example, have a critical problem of light crossover between adjacent subpixels, due to the presence of color filters. However, our color-filterless, stacked configuration may solve this problem, through the use of highly transparent monolayered-QD OQO phototransistors. These devices can also be applied to high-resolution image sensors without lateral subpixels for colors, which is made possible by the realization of this vertically stacked RGB-tandem structure.

### Noise and detectivity

**Fig. 6:**
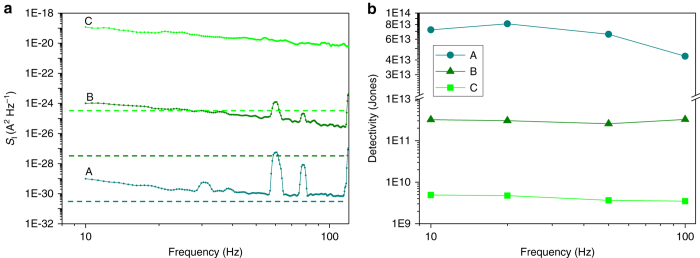
Noise power spectral density and specific detectivity. **a** *S*_I_ as function of modulation frequency in 1GQ-OQO phototransistor. A, B, and C denote three different measurement regimes, corresponding to *V*_G_ values of −18, −15, and 12 V, respectively, (see also, Supplementary Fig. 13a). **b** Estimation of D* for three regimes of operation vs. modulation frequency

On the other hand, noise is another important figure of merit that must be carefully characterized. Therefore, we measured the noise power spectral density (*S*_I_) and D*, as shown in Fig. 6, using an SR570 low-noise current amplifier and a HP89441 vector signal analyzer. The OQO phototransistors for noise measurement and D* were designed to have a square shape (50 × 50 μm^2^). Among the various kinds of noise, shot noise, Johnson noise, and 1/*f* noise are dominant in phototransistors^5, 10, 32, 33^. The shot noise limit is expressed as $< I_{{\rm{SN}}}^2  >= 2q{I_{\rm{D}}}\Delta f$ and the Johnson noise is $4kT\Delta f{\rm{/}}{R_{\rm{D}}}$, where *q* is the electron charge, *I*_D_ is the dark current, Δ*f* is the noise bandwidth, *k* is the Boltzmann constant, *T* is temperature, and *R*_D_ is the detector resistance under dark conditions. We chose three different *V*_G_ values representing the subthreshold (A), ohmic (B), and near-saturation (C) regimes (see Supplementary Fig. 13a).

In Fig. 6a, the flicker 1/*f* noise, *S*_*I*_~1/*f* increases with respect to increasing drain current levels, as indicated by A, B, and C for the 1GQ-OQO phototransistors. The shot noise limit at each current level is also plotted in dotted lines. We do not consider the Johnson noise, because this component is one order of magnitude lower than the shot noise in our devices (see Supplementary Note 7). In the subthreshold regime (A), the measured *S*_I_ is closer to the shot noise limit at high frequencies of modulation than in other operating regimes. This implies that we can achieve extremely low noise levels by controlling *V*_G_, which is a unique advantage of phototransistors. Based on this noise analysis, the photodetector sensitivity can be explicitly expressed using the D* parameter. D* can be rewritten as ${D^*} = \sqrt {A\Delta f} {\rm{/NEP}} = R\sqrt {A{\rm{/}}{S_{\rm I}}}$, where *A* is the effective area of the detector in cm^2^ and *R* is in AW^−1^, measured under the same conditions as *S*_I_ (see more details in Methods and Supplementary Fig. 13b). Figure 6b represents the calculated results for D* vs. the modulation frequency with respect to A, B, and C. An inverse relationship between D* and *I*_*D*_ is observed, as expected. The D* of the 1GQ-OQO phototransistor reaches 8.1 × 10^13^ Jones at 20-Hz frequency in regime A. Such ultrasensitive photodetection can be attributed to the proposed OQO configuration with monolayered QDs, which facilitates effective photocarrier control. This D* is approximately 17% higher than the previous record for QD-graphene hybrid phototransistors (7 × 10^13^ Jones)^11^. Moreover, D* can be further enhanced by reducing the flicker 1/*f* noise, when the surface defects of QDs are controlled to reduce trap sites or defects in the channel.^10^

## Discussion

We demonstrated ultrasensitive photodetection using monolayered QDs buried in amorphous-oxide SIZO phototransistors. The effectively large interfacial areas of the QDs buried in the OQO configuration facilitates the observation of fast charge separation and charge transport caused by the high-mobility SIZO layers. Hence, we achieved RGB-color detection exhibiting a *G*_meas_ value as high as 10^7^, which is obtained via room-temperature deposition of the SIZO layers. By combining the transparent and color-sensitive characteristics, a logic circuit for color-selective detection was successfully demonstrated using vertically stacked multi-color OQO phototransistors without color filters. The concept proposed in this study also allows monolithic integration with a CMOS-compatible process, which presents a basis for myriad emerging applications in color detection with sensitivity, transparency, and even flexibility.

## Methods

### Device fabrication

We synthesized three different species of red (CdSe), green (CdSeS), and blue (CdS) QDs, of approximately 4.5 nm in diameter. More details of the QD synthesis and purification are presented in the Supplementary Method 1. Amorphous SIZO TFTs with a bottom gate and top electrodes were fabricated using conventional photolithography. The bottom amorphous SIZO film was deposited on a SiO_2_ (200 nm)/Si substrate using room-temperature RF sputtering at 0.56-Pa pressure in mixed Ar/O_2_ gases. After spin-coating of the QDs, the top amorphous SIZO film was deposited under the same conditions as for the bottom SIZO film deposition. Then, a low-temperature, annealing process was conducted at 150 °C. The bottom and top SIZO layers had thicknesses of 40 and 30 nm, respectively. The channel widths and lengths were 250 and 50 μm, respectively, expect for OQO phototransistors for noise and detectivity measurement. Regarding the thickness of QD layers, we controlled the concentration of QD solution and the speed of spin coating. For example, 1 wt% QD in cyclohexane solution with a coating speed at 2000 rpm was applied for six-layered QD films.

### Photodetection measurement

The photodetection measurement was conducted using a custom-built confocal scanning microscope. All the measurements were performed using monochromatic solid-state CW diode lasers of 487- and 403-nm wavelength except for spectral R (responsivity) measurement as shown in Fig. 2c. For such spectral R measurements (Fig. 2c) together with logic-inverter measurement (Fig. 5), an optical parametric generator-amplifier (OPG-OPA) pulse laser (Opolett HR355, OPOTEK) was used as a tunable light source (400–700 nm). The spatial photocurrent images in Fig. 1b were obtained using laser beam scanning through an objective lens (×50, 0.8 numerical aperture). The objective lens of the microscope focused the beam to a diffraction-limited spot of 500 nm in diameter on the device. The source-drain voltage is 5 V for all the measurement, i.e., transfer curves, noise measurement, etc.

### Detectivity measurement

The detectivity is related to the NEP by following expression: ${D^{\rm{*}}} = \sqrt {A\,\Delta f} {\rm{/NEP}}$, where A is the photosensitive area of the device and Δ*f* is the detection bandwidth. The NEP is defined as the amount of incident light power inducing a photocurrent equal to the noise current, which is given by: ${\rm{NEP}} = \sqrt {\left\langle {I_{\rm{n}}^2} \right\rangle } {\rm{/}}R = \sqrt {{S_{\rm{I}}}\Delta f} {\rm{/}}R$, where $\left\langle {{{I}}_{\rm{n}}^2} \right\rangle$ is the time-averaged square of the total noise current and *R* is the responsivity. Here, we measured the noise spectral density (*S*_I_) of the drain current under dark conditions using an SR570 low-noise current amplifier and a Hewlett Packard 89441 vector signal analyzer. The measurement was conducted in an electrically and optically shielded probe station. The OQO phototransistors for noise measurement were designed to have a square shape (50 × 50 μm^2^) photosensitive area (*A*).

### Data availability

The data that support the findings of this study are available from the corresponding authors on request.

## Electronic supplementary material

Supplementary Information
