# Supplementary material for: Color-selective photodetection from intermediate colloidal quantum dots buried in amorphous-oxide semiconductors
Source: Nat Commun. 2017 Oct 10;8:840. doi: 10.1038/s41467-017-00893-x (PMC5635032; doi:10.1038/s41467-017-00893-x)
Supplement: Supplementary file 1 — Supplementary Information [file 41467_2017_893_MOESM1_ESM.pdf]

File name: Supplementary Information
Description: Supplementary Methods, Supplementary Figures, Supplementary Tables and Supplementary Notes

SUPPLEMENTARY METHODS

Supplementary Method 1. QD synthesis and purification

Colloidal QDs for RGB colors were synthesized using wet chemical methods. The red, green, and blue QDs had similar sizes (~4.5 nm), but different compositions, i.e., CdSe, CdSeS, and CdS, respectively. The red QDs (CdSe) were synthesized following the procedure reported in previous publications (ref). Briefly, 1.6-mmol CdO powder (0.206 g, Aldrich, +99.99 %) and 6.4-mmol oleic acid (OA, 1.8 g, Aldrich, 95 %) were mixed with 40-mL trioctylamine (TOA, Aldrich, 90 %). The mixed solution was degassed and heated to 150 ℃ under rapid stirring, and the temperature was then further increased to 300 ℃ under an N_2 flow. At 300 ℃, 0.2-mL 2.0M Se (Alfa) in trioctylphosphine (TOP, Strem, 97 %) was quickly injected into the Cd-containing reaction mixture. The total growth time was 3 min. The green QDs (CdSeS) were synthesized by injecting mixed Se and S precursors into a hot solution of Cd precursors under Cd excess conditions (ref). Then, 0.1-g CdO, 0.92-g oleic acid, and 32-ml trioctylamine (TOA) were heated to 300 ℃ under an N_2 flow, yielding a colorless solution. After the solution temperature was maintained steadily at 300 ℃, a 2-ml premixed (1.94-ml Se and 0.06-ml S) 1M trioctylphosphine solution was injected rapidly into the CdO/OA/TOA solution with vigorous stirring and reacted for 3 min. For the blue QDs (CdS), 2 ml of S was used instead of the premixed solution. After the reaction, the product was cooled to ca. 50–60 ℃, and the organic sludge was removed during the first centrifugal separation (6,000 rpm). Ethanol (Fisher, HPLC grade) was added to the product solution until an opaque flocculant appeared, and the nanocrystals were separated by centrifugation. The precipitates were dispersed in toluene (Sigma–Aldrich, anhydrous 99.8 %) to be characterized with a UV-vis spectrometer (Varian Cary 5000) and a fluorometer (Fluorolog JOBIN YVON Horiba).

Supplementary Method 2. Calculation of charge separation rate in OQO devices

The kinetics of the excited QD population ($N_{\mathrm{QD}^*}(t)$) can be simply expressed by

$$N_{\mathrm{QD}^*}(t) = ae^{-kt}, \qquad \text{(Supplementary Equation 1)}$$

where a is a coefficient and k is a decay rate. This can be resolved into the dynamics of a photocarrier decay rate (k_{QD}) in QDs and a charge separation rate ($k_{\mathrm{CS,\,OQO}}$) from QDs to SIZO layers in OQO configuration as follows:

$$k_{\mathrm{OQO}} = k_{\mathrm{QD}} + k_{\mathrm{CS,\,OQO}}. \qquad \text{(Supplementary Equation 2)}$$

Here, we can easily obtain the charge separation time ($\tau_{\mathrm{CS,\,OQO}} = 1/k_{\mathrm{CS,\,OQO}}$) expressed by

$$\frac{1}{\tau_{\mathrm{OQO}}} = \frac{1}{\tau_{\mathrm{QD}}} + \frac{1}{\tau_{\mathrm{CS,\,OQO}}}, \qquad \text{(Supplementary Equation 3)}$$

where τ_{OQO} is 0.29 ns and τ_{QD} is 7.7 ns from the fitting curves as indicated by green and black solid lines in Fig. 3a, respectively. Therefore, we can extract the charge separation time ($\tau_{\mathrm{CS,\,OQO}}$) of 300 ps in OQO configuration, corresponding to the charge separation rate ($k_{\mathrm{CS,\,OQO}}$) of 3.3 ns^{-1}. In the same way, we can extract the charge separation time ($\tau_{\mathrm{CS,\,QO}}$) of 2 ns in QO configuration, corresponding to the charge separation rate ($k_{\mathrm{CS,\,QO}}$) of 0.5 ns^{-1} in QD-on-SIZO films from τ_{QO} of 1.6 ns and τ_{QD} of 7.7 ns, according to the following relations:

$$k_{\mathrm{QO}} = k_{\mathrm{QD}} + k_{\mathrm{CS,\,QO}} \qquad \text{(Supplementary Equation 4)}$$

and

$$\frac{1}{\tau_{\mathrm{QO}}} = \frac{1}{\tau_{\mathrm{QD}}} + \frac{1}{\tau_{\mathrm{CS,\,QO}}}. \qquad \text{(Supplementary Equation 5)}$$

Therefore, the charge separation rate of OQO films is more than six times that of the QD-on-SIZO films.

SUPPLEMENTARY FIGURES

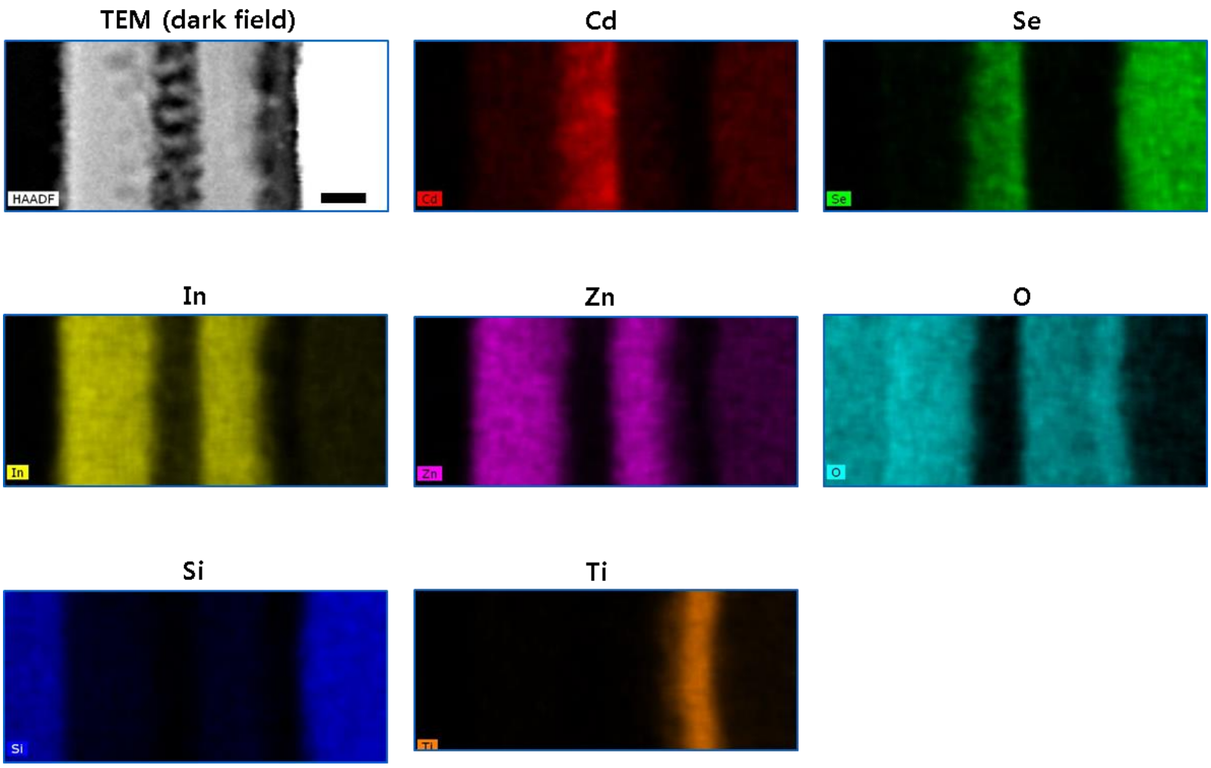

Supplementary Figure 1. EDS analysis of OQO phototransistor in figure 1a in the text. Scale bar is 50 nm.

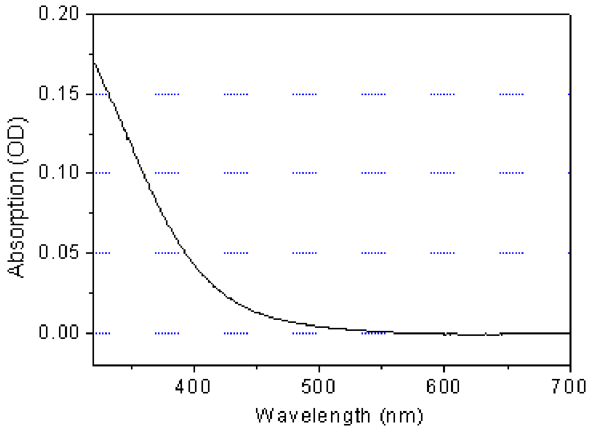

Supplementary Figure 2. UV-vis absorption of 40 nm SIZO film on Quartz.

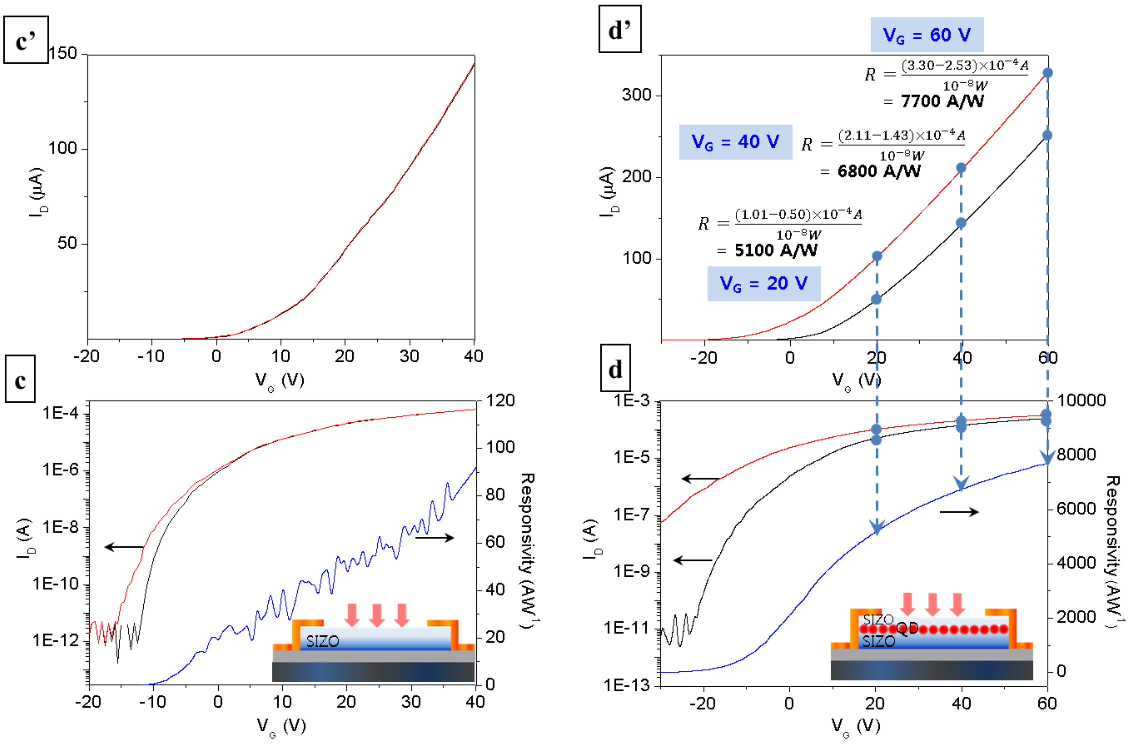

Supplementary Figure 3. Replots of Fig. 1c and 1d for responsivity calculation in linear scale as shown in c' and d', respectively.

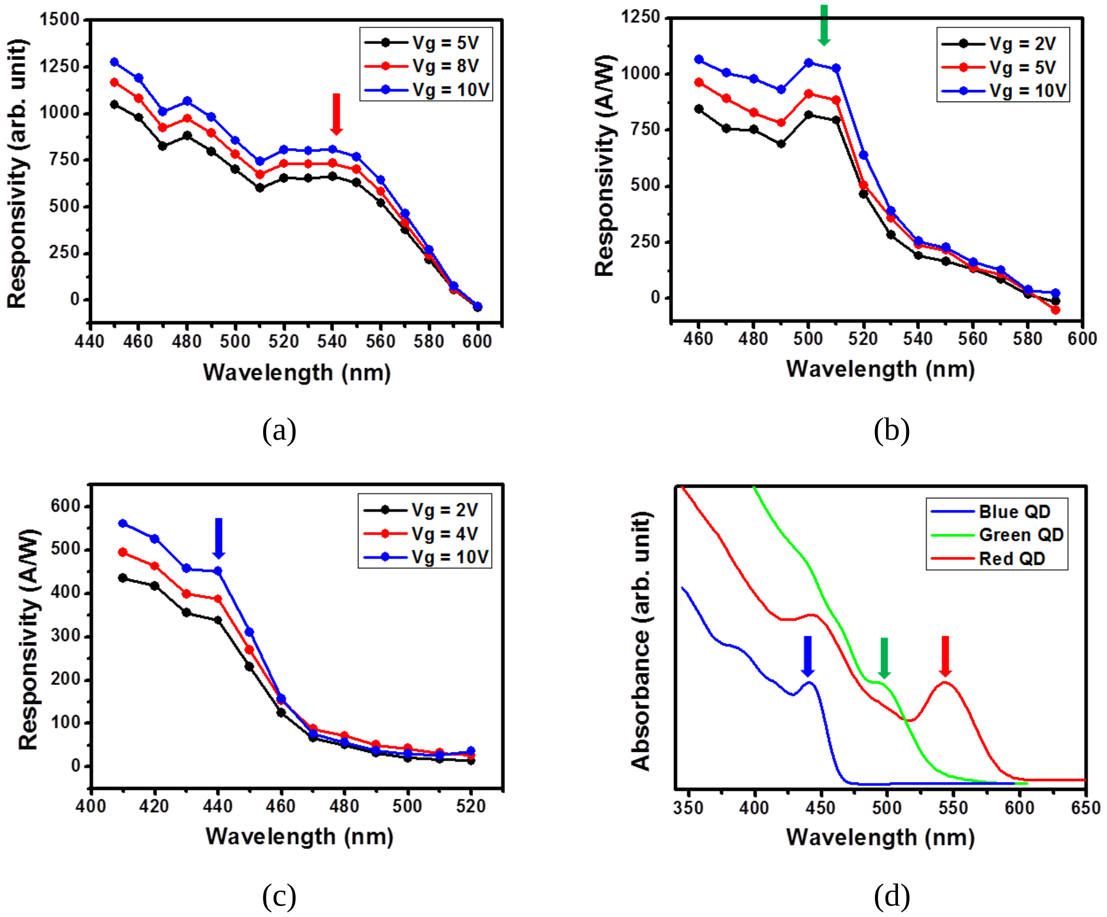

Supplementary Figure 4. Spectral *R* of OQO devices with respect to V_G. (a) 4RQ-OQO (b) 6GQ-OQO (c) 6BQ-OQO. (d) Absorption spectra of R, G, and B QDs.

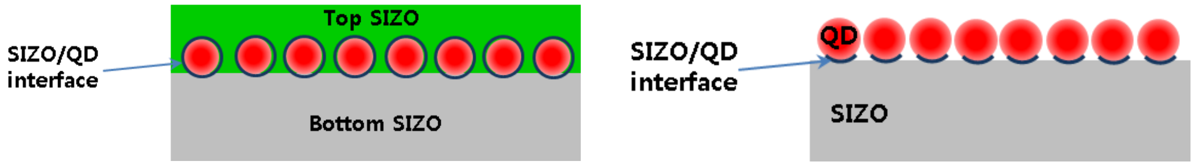

(a) (b)

Supplementary Figure 5. Schematics of the (a) OQO and (b) QO device configuration.

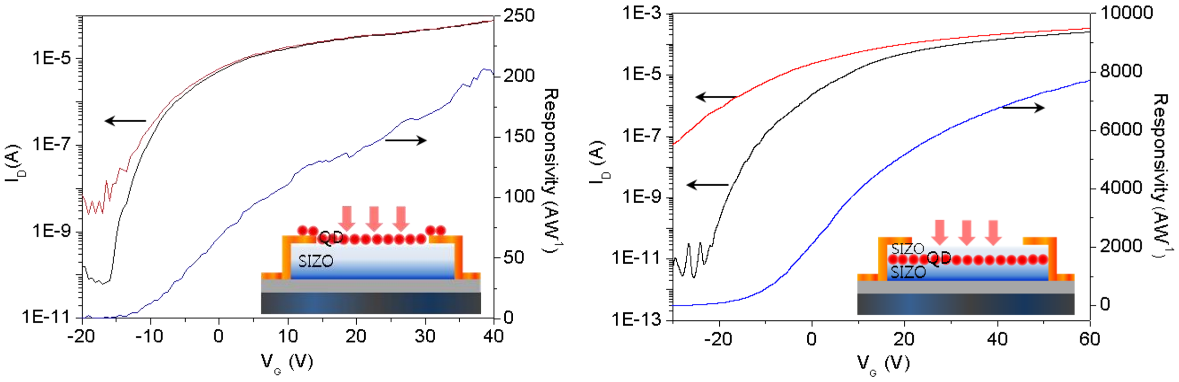

(a) (b)

Supplementary Figure 6. Photocurrent responses and reponsivities of phototransistors with monolayered red QDs placed on top of SIZO layer (1RQ-QO structure) and (b) monolayered red QDs buried in SIZO layers (1RQ- OQO structure) at 487-nm illumination wavelength with 10-nW power. Black and red lines are dark current and photocurrent, respectively. Responsivities are indicated by blue lines.

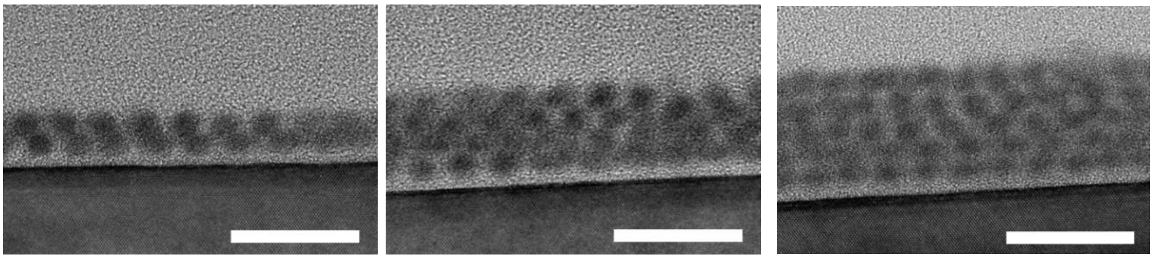

(a) (b) (c)

Supplementary Figure 7. TEM image of various multi-layered R-QD films. (a) 2-layer, (b) 4-layer, (c) 5 layer. The scale bar is 30 nm.

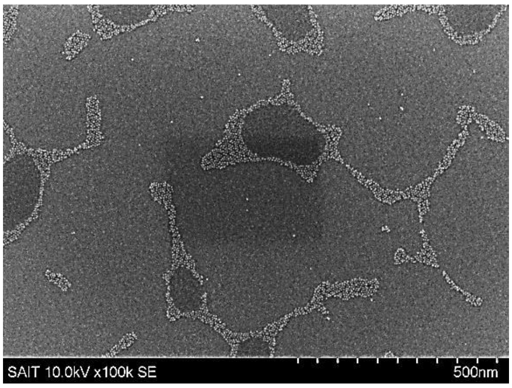

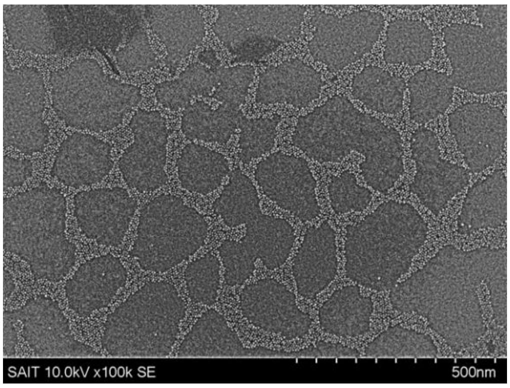

(a) **(b)**

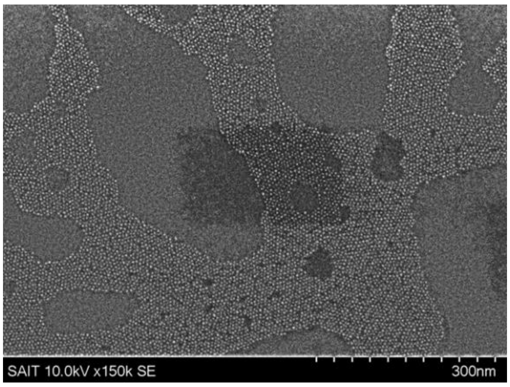

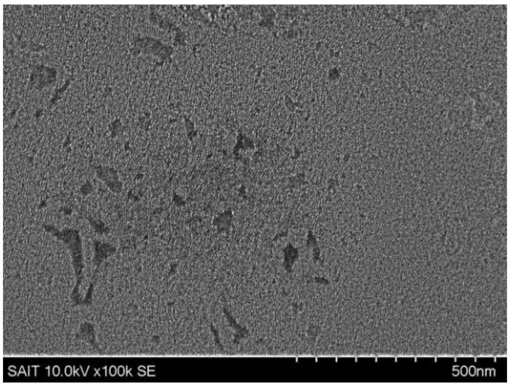

(c) **(d)**

Supplementary Figure 8. SEM image of various R-QD films (top view). (a)0.1 L, (b) 0.2 L, (c) 0.5 L, (d) 1 L. The areal coverage of QDs are 9.3% (0.1 L), 23.1% (0.2 L), 52.2% (0.5 L), and 96.5% (1 L), respectively.

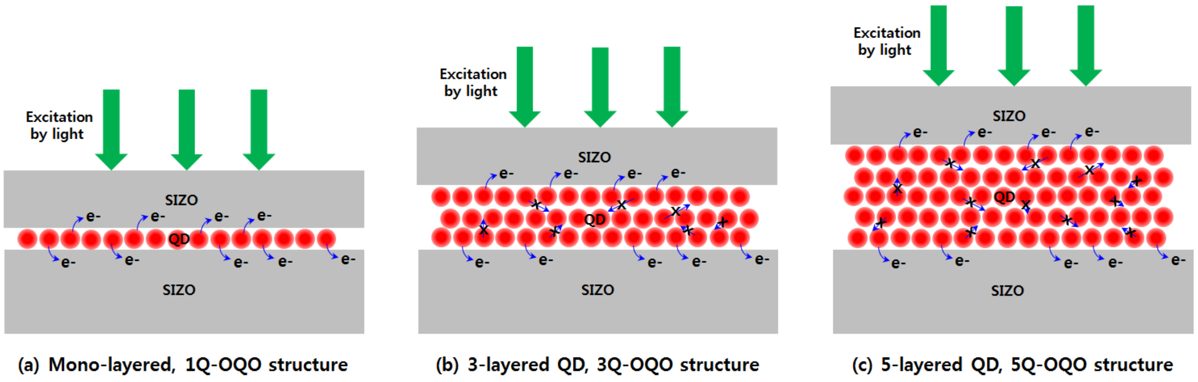

Supplementary Figure 9. Comparison of different QD-thickness structures

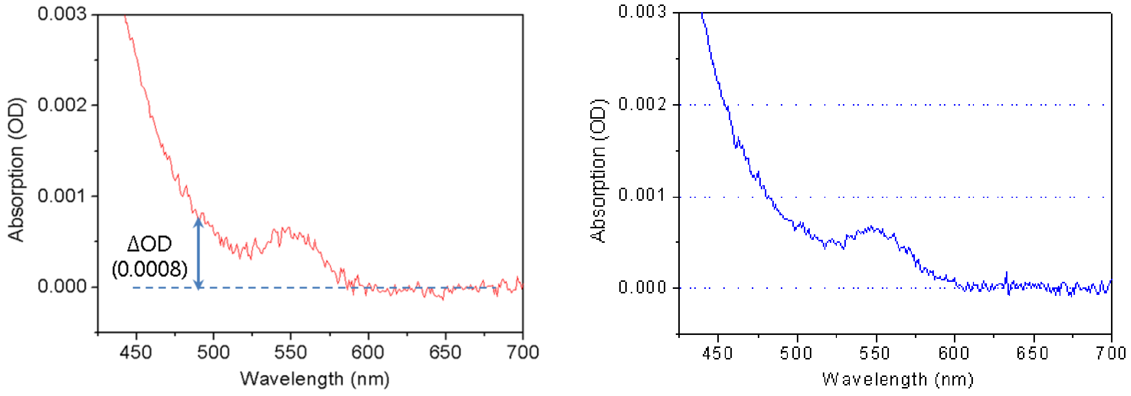

(a) (b)

Supplementary Figure 10. Absorption spectra of (a) 1RQ-OQO film and (b) the red-monolayered QD film on Quartz obtained using $1 - R_{\text{light}} - T_{\text{light}}$. These two spectra have little difference, which means there is negligible QD damages after top SIZO layer deposition in 1RQ-OQO film.

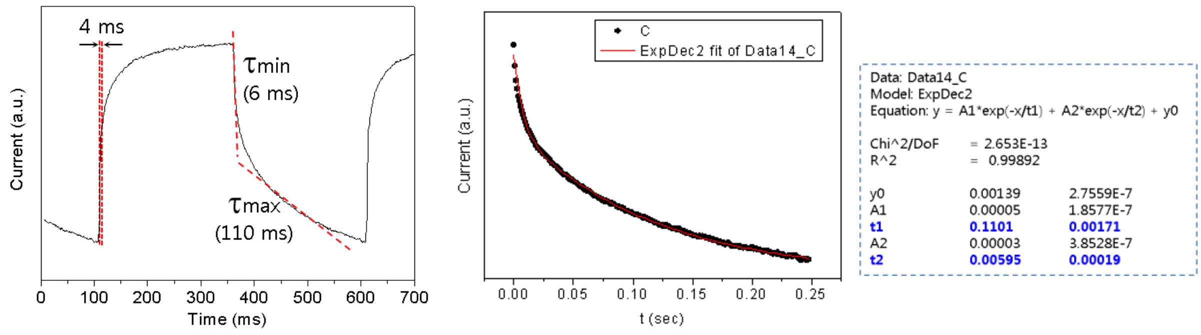

(a) (b)

Supplementary Figure 11. Temporal photocurrent response of 1RQ-OQO devices at 487-nm wavelength with 5-μW input power. (a) The temporal response indicates a rise time of 4 ms and two different fall times of 6 and 110 ms. (b) The extraction of decay time constants using a two-exponential function.

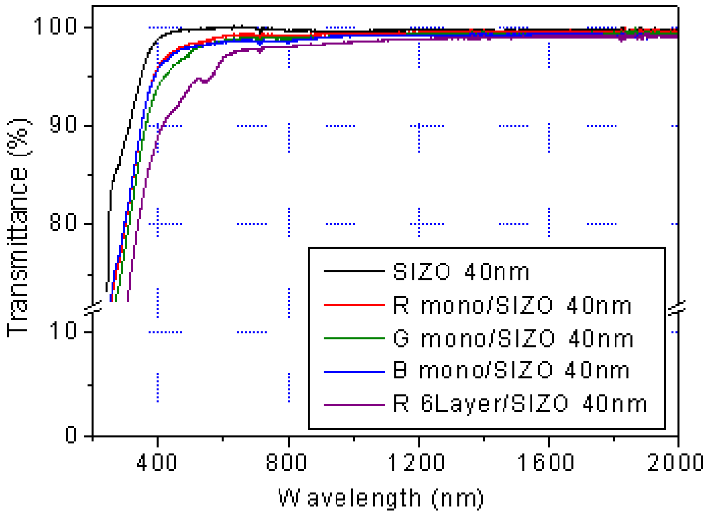

Supplementary Figure 12. Transparencies of various SIZO and QD films.

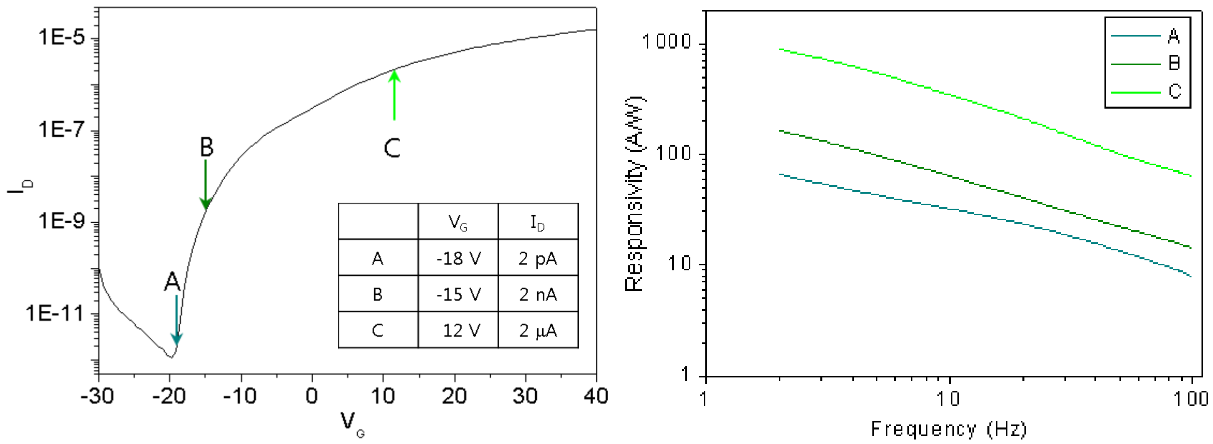

(a) (b)

Supplementary Figure 13. Transfer curve and *R* of 1GQ-OQO device with 50 μm × 50 μm channel area. (a) Device output characteristics. A, B and C denote the points where V_G = -18, -15, and 12 V, respectively. The output currents at each V_G for A, B, and C are listed in the inset table. The three different V_G represent the subthreshold (A), ohmic (B), and near-saturation (C) regimes, and the corresponding frequency-modulated responsivities are shown in (b).

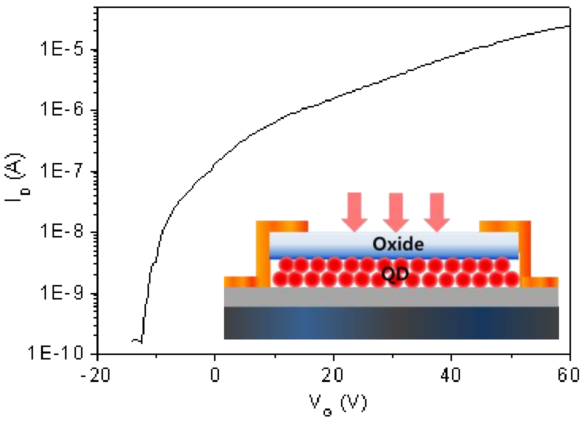

Supplementary Figure 14. Measured I-V transfer curve for OQ phototransistors with a 40-nm thick SIZO layer on top of 6-layered QDs.

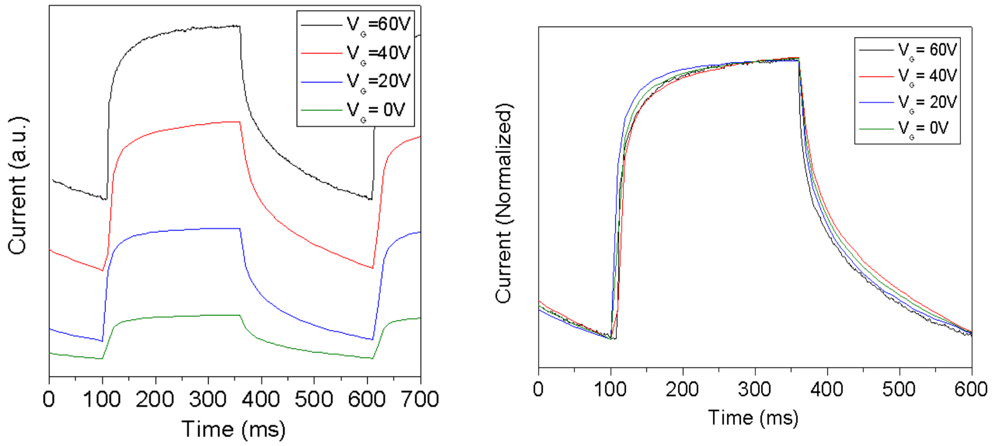

(a) **(b)**

Supplementary Figure 15. (a)Temporal photocurrent responses of the 1RQ-OQO device with different gate voltages (V_G) at 487-nm wavelength and 5-μW light power. (b) Each curve in (a) is normalized by its minimum and maximum values. The rise and decay time constants do not change as V_G varies.

SUPPLEMENTARY TABLES

Supplementary Table 1. Electrical characteristics and field-effect mobilities of single-layered SIZO device and OQO structures with various QD layer types under dark condition.

Device	V_{th}	I_{on}	I_{off}	On/Off	FE mobility
SIZO only, 40 nm	1.9	1.2.E-04	-2.1.E-13	5.5.E+08	15.6
Red QD, monolayer OQO	-2.0	1.7.E-04	7.8.E-12	2.1.E+07	14.5
Red QD, 2-layer OQO	-5.7	2.1.E-04	-2.2.E-11	9.3.E+06	15.7
Red QD, 4-layer OQO	-4.6	3.9.E-04	-5.0.E-09	7.7.E+04	12.5
Red QD, 6-layer OQO	-7.4	1.3.E-04	1.2.E-11	1.1.E+07	16.4
Red QD, 9-layer OQO	-11.3	8.8.E-05	3.8.E-11	2.3.E+06	9.6
Green QD, monolayer OQO	-6.5	5.8.E-05	1.6.E-11	3.6.E+06	13.3
Green QD, 6-layer OQO	-5.4	1.0.E-04	1.0.E-11	9.9.E+06	9.9
Blue QD, monolayer OQO	-1.5	1.1.E-04	-3.8.E-11	2.8.E+06	10.8
Blue QD, 6-layer OQO	-6.9	1.7.E-04	2.1.E-11	8.3.E+06	14.3

The electrical characteristics of a single-layered SIZO device and OQO structures with various QD layer types are presented in Supplementary Table S1. The field-effect mobilities range from 9.6–16.4 $cm^2V^{-1}s^{-1}$. They do not show strong dependence on the species or thicknesses of the QD layers.

Supplementary Table 2. Temporal response of decay time for various illuminating light powers.

Input power	τ_1 (ms)	τ_2 (ms)
200 nW	70	2500
1 μW	31	400
5 μW	6	110

SUPPLEMENTARY NOTES

Supplementary Note 1. Comparison of interface area between QD and SIZO

As shown in Supplementary Fig. 5, the contact interfacial area between QDs and oxides in OQO structure (Supplementary Fig. 5a) should be much larger (more than 2 times) than QO configuration (Supplementary Fig. 5b), because the top and bottom oxide layers surround all the surface of QDs. This is provided by the RF sputtering of top SIZO layers allowing conformal deposition and close contact between QDs and oxides. On the other hand, the contact area in QO devices is only a fraction of the surface of QDs, which can hardly be a half of the QD surface area due to the spin coating of QDs on the flat, rigid surface of oxide layer. Thus, this difference in device configuration has led to the observation of about 6 times of the charge separation rate for the sandwich-like OQO structure compared with QO devices. This is one of the most important advantages, which characterizes the unique property of our proposed OQO configuration.

Supplementary Note 2. Effect of QD thickness on charge generation and flow in OQO device.

As shown in Supplementary Fig. 9, the characteristics of charge generation and flow are compared in three different QD-thickness structures. Firstly, the number of trap sites increases as the thickness of QD layers increases. That is, there is a larger number of dangling bonds in thicker QD films, which mainly interrupts efficient charge flow. Secondly, the effective mean free path (l_{eff}) of an electron from nano-sized QDs is given by

$$\frac{1}{l_{\mathrm{eff}}} = \frac{1}{r} + \frac{1}{l_{\mathrm{f}}} , \qquad \text{(Supplementary Equation 6)}$$

where r is the radius of QDs and l_f is the mean free path in bulk[30]. Considering CdSe, l_{eff} becomes only 2.2 nm, because l_f is about 20 nm in bulk and r is around 2.5 nm. This is comparable to the size of QDs so that photoexcited charges in the middle of QD layers are going to experience serious collisions even in the 3Q-OQO structure. Thirdly, the effective field strength given by the gate voltage becomes weak due to the increase of gap distance between source and gate electrodes.

Supplementary Note 3. Calculation of saturable light power for mono-layer OQO devices

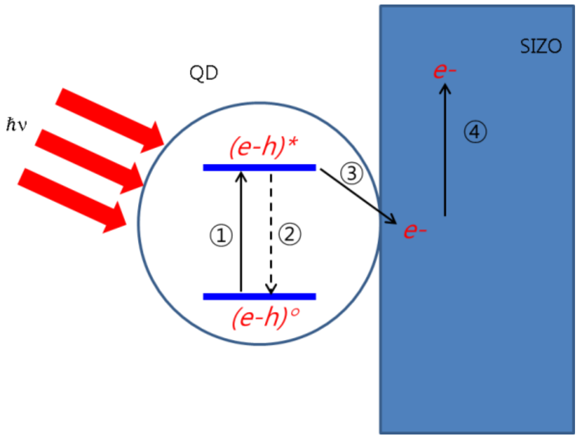

Supplementary Figure 16. , Charge transport mechanism. (1) exciton generation (~a few fs), (2) exciton recombination (τ_{CR} ~7.7 ns), (3) charge separation (τ_{CS} ~ 300 ps) from QDs to SIZO, and (4) charge transport through SIZO.

The schematics of charge transport mechanism in OQO devices is shown in the figure above. After light absorption, there are typical photocarrier processes such as: (1) exciton generation, (2) exciton recombination (radiative or non-radiative), (3) charge separation from QDs to SIZO, and (4) charge transport through SIZO. Thus, the saturable light power can be decided by the competition among the following three processes: absorption saturation (no

more light absorption by empty $(e\text{-}h)^o$ ground state), charge separation, and charge transport through SIZO. And we think the absorption saturation may not be the rate-limiting step because the light intensity needed for absorption saturation is very high.

The absorption saturation was explained the bleaching of the excitonic transitions (ref. J. Chem. Phys. 116(9)(2002)3828-3833). In the pulse laser excitation, the light power for saturation of the bleaching can be obtained when every single QD receives a single photon. The calculation of saturable light power is as follows:

- Energy of single photon at 487 nm: 4.07×10^{-19} J
- Packing density of QD: $10^{12} \sim 10^{13}$ cm^{-2}

Considering CW light illumination at saturation, the repetition of photons should be equal to the decay lifetime of exciton (e-h)*. When we think about the lower and upper limits of exciton lifetime, the longest exciton lifetime can be the single QD fluorescence lifetime in diluted solutions or dispersed matrices. In diluted systems, for example, the previous work reports the exciton lifetime of 25 ns [Ref. JPCB 108, 143 (2004)]. On the contrary, we may take into consideration of the shortest exciton lifetime in our OQO devices as fast as the measured charge separation time of 300 ps. If the lifetime is less than 300 ps, the charge separation will not occur because the recombination process dominates. Thus, the lower limit of exciton lifetime is 300 ps.

Now we estimate the lower and upper bounds of saturable light intensity as follows:

Saturable light intensity = (Energy of single photon at 487 nm)

$\times$ (Packing density of QDs)

$\times$ (Number of photons required for exciton generation in unit time)

$= (4.07 \times 10^{-19}$ J) x $(10^{12} \sim 10^{13}$ cm^{-2}) x ($\frac{1}{20}$ ns$^{-1} \sim \frac{1}{300}$ ps^{-1})

$= 20.4 \sim 1.34 \times 10^{4}$ J cm^{-2}s^{-1} = 20.4~1.34×10^{4} Wcm^{-2}

When we consider the beam spot size (diameter of 50 μm),

Saturable light power $= (20.4 \sim 1.34 \times 10^{4}$ Wcm$^{-2}) \times (1.96 \times 10^{-5}cm^{2})$

$= 400\ \mu$W~2.63 W

Thus, light power we used (5 μW) is still far from the absorption saturation regime.

In the QO phototransistor in [Ref 7], the absorption saturation might be similar to the above calculated values. But in the QO phototransistor, the saturation of photocurrent occurred at the light power intensity of 300 μW/cm^2, which mean the charge separation (τ_{QO}) is very slow, compared with our OQO configuration. We investigated this value as 18.1 ns ($1/\tau_{QO} = 1/\tau_{QD} + 1/\tau_{CS,\,QO}$), where $\tau_{QO} = 4.42$ ns and $\tau_{QD} = 5.85$ ns.

In contrast, the charge saturation in our phototransistors was not observed even at high intensity of light power (255-mW/cm^2) and this is one of the advantages of fast charge separation in OQO configuration.

Supplementary Note 4. Inherent photoconductive gain

We employ the new concept of G_{inher}, including net absorption in the OQO channels, by eliminating the device R_{light} and T_{light}. The red curve shown in Supplementary Fig. 10a is the net absorptions of the 1RQ-OQO channel. The absorbance at 487 nm is 0.0008, which indicates 0.02% light absorption. Thus, G_{inher} is 500 times larger than G_{meas}, as shown by the blue and red curves in Fig. 3c, respectively.

Supplementary Note 5. Photoconductive gain using photocurrent-response decay times

Supplementary Fig. 11 shows the temporal response of the monolayered-red QD OQO phototransistors for 5-μW laser power at 487-nm wavelength with a 2-Hz repetition rate. The rise time is 4 ms and the decay time has two components, i.e., fast and slow decay times of 6 and 110 ms, respectively. From the measured photoresponse time, the calculated photoconductive gain is in the 1.2×10^4–2.2×10^5 range; this is according to the equation, $G_\tau = \tau_{\text{lifetime}}/\tau_{\text{transit}}$, where the τ_{transit} of the OQO films is 500 ns. The temporal responses for two other light powers of 200 nW and 1 μW were also measured. The decay lifetimes are summarized in Supplementary Table 2 and the corresponding photoconductive gains ($G_{\tau\text{min}}$ and $G_{\tau\text{max}}$) are marked in Fig. 3c.

Supplementary Note 6. Transparencies of SIZO and QO films

The transparencies of the SIZO-only and the QD-on-SIZO films are shown in Supplementary Fig. 12. The transparency depends on the QD layer thickness. The 6-layered-red-QD (6RQ)-on-SIZO film exhibits the lowest transparency of 90% at 400-nm wavelength. In contrast, the films with monolayered QDs on SIZO for the different RGB colors exhibit higher transparencies of more than 95% at the same wavelength.

Supplementary Note 7. Johnson noise calculation

The Johnson noise is $4kT\Delta f/R$, where k is the Boltzmann constant, T is the temperature, Δf is the noise bandwidth, and R is the detector resistance under dark conditions. In the subthreshold regime, A (V_G = -15V), R is ca. 10^{12} Ω; therefore, the Johnson noise is 1.6 x 10^{-32} A^2Hz^{-1}. In the ohmic (B) and near-saturation regimes (C), the Johnson noise becomes 1.6 x 10^{-29} and 1.6 x 10^{-26} A^2Hz^{-1}, respectively.

Supplementary Note 8. Effect of field screening by QDs

In our OQO phototransistors, the thickness of QD layers is less than a few ten of nanometers, e.g., 30 nm for 6-layered QD films. Thus, the field screening effect of QDs is considered small that top SIZO layers play a role as a charge transport layer. For example, we prepared OQ phototransistors with a 40-nm oxide layer on top of 6-layered QDs and investigated whether the top oxide layer can support charge flows, as depicted in Supplementary Fig. 14. It is evidently shown that even the oxide channel layer on top of 30-nm thick, 6-layered QDs can be successfully gated by V_G.
